# Mung Bean (*Vigna radiata* L.) Source Leaf Adaptation to Shading Stress Affects Not Only Photosynthetic Physiology Metabolism but Also Control of Key Gene Expression

**DOI:** 10.3389/fpls.2022.753264

**Published:** 2022-02-04

**Authors:** Xiangwei Gong, Chunjuan Liu, Ke Dang, Honglu Wang, Wanli Du, Hua Qi, Ying Jiang, Baili Feng

**Affiliations:** ^1^College of Agronomy, Shenyang Agricultural University, Shenyang, China; ^2^State Key Laboratory of Crop Stress Biology in Arid Areas, College of Agronomy, Northwest A&F University, Yangling, China

**Keywords:** gene expression, mung bean, photosynthesis, plant hormones, shading stress

## Abstract

Shading stress strongly limits the effective growth of plants. Understanding how plant morphogenesis and physiological adaptation are generated in response to the reduced low light conditions is important for food crop development. In this study, two mung bean (*Vigna radiata* L.) cultivars, namely, Xilv 1 and Yulv 1, were grown in the field to explore the effects of shading stress on their growth. The results of morphology, physiology, and biochemistry analyses showed that the shading stress significantly weakened the leaf photosynthetic capacity as measured by the decreased net photosynthetic rate, stomatal conductance, and transpiration rate and increased intercellular CO_2_ concentration. These responses resulted in plant morphological characteristics that increased the light energy absorption in low light conditions. Such variations occurred due to the leaf anatomical structure with destroyed palisade tissues and spongy tissues. Under shading stress, Yulv 1 showed higher physiological metabolic intensity than Xilv 1, which was related to changes in chlorophyll (Chl), such as Chl *a* and *b*, and Chl *a/b* ratio. Compared with normal light conditions, the Chl fluorescence values, photosynthetic assimilation substances, and enzyme activities in mung bean plants under shading stress were reduced to different extent. In addition, the relative expression levels of *VrGA2ox*, *VrGA20ox1*, *VrGA3ox1*, *VrROT3*, and *VrBZR1*, which are related to endogenous hormone in mung bean leaves, were upregulated by shading stress, further leading to the improvements in the concentrations of auxin, gibberellins (GAs), and brassinolide (BR). Combined with the morphological, physiological, and molecular responses, Yulv 1 has stronger tolerance and ecological adaptability to shading stress than Xilv 1. Therefore, our study provides insights into the agronomic traits and gene expressions of mung bean cultivars to enhance their adaptability to the shading stress.

## Introduction

The shading stress or weak light condition is one of the common abiotic stresses in agricultural production, which restrains the growth of plants in some unfavorable cultivation practices, such as high planting density (Liu et al., 2010) and agroforestry compound systems (Zhang et al., 2018). The leaves are the main organs of plant photosynthesis that first detect changes in the light intensity and quality. As a photoautotrophic organism, plant leaf produces carbohydrates through photosynthesis to provide energy for their own growth and development. Therefore, when the external ecological environment changes, the photosynthetic capacity of leaves is directly and significantly affected (Dai et al., 2009; Liu et al., 2017). Many studies have indicated that shading seriously reduces the leaf assimilate supply and decreases the light energy absorption by blocking the electron flow rate from photosystem II (PSII) to photosystem I (PSI) and ATP synthesis (Terashima et al., 2006; Zivcak et al., 2014). At present, there are two main mechanisms for the reduction in photosynthesis: (i) The diffusion of CO_2_ in leaves decreases, which is due to the decline of intercellular CO_2_ and stomatal conductance; and (ii) inhibiting the metabolic potential of photosynthesis by controlling cell proliferation and the growth and expansion of leaves (Wu et al., 2017; Yao et al., 2017b). Of course, different crop cultivars have different photosynthetic and physiological responses to the same shading environment. For instance, Yao et al. (2017a) reported that compared with the shading-sensitive soybean cultivar L29, cultivar L32 exhibits higher photosynthetic characteristics and productivity attributed to PSII activity and energy transport from PSII to PSI under shading stress. Wang et al. (2010) found that Zhongza 9 was more resistant than Zhongshu 6 under weak light condition, as indicated by the photosynthetic characteristics and chlorophyll (Chl) fluorescence parameters. Therefore, to a certain extent, elucidating the mechanisms underlying shading avoidance of plants is essential for breeding shade-tolerant cultivars and improving plant production.

The effect of shading on plant performance has been widely studied. Overall, plant leaves in shade environment have lower net CO_2_ assimilation rate and electron transfer carriers than those in the natural condition (Zhang et al., 2004; Tateno and Taneda, 2007). Under this condition, the net photosynthetic rate would be the major factor driving crop carbon balance, concentration of light capturing components, and activity of energy-transferring enzymes (Yang et al., 2018a). During photosynthesis, ribulose-1,5-bisphosphate carboxylase (Rubisco) regulates CO_2_ fixation (Ranjbarfordoei et al., 2006), which directly participates in the primary processes of photosynthesis in the Calvin cycle. Consequently, the main physiological restraint involved in shading-associated downregulation of net photosynthetic rate is the decline of Rubisco activity. In addition, shading stress at the vegetative stage severely modulates the concentration of thylakoid components and number of reaction centers (Kunderlikova et al., 2016), as well as the level of PSII and the limitations in electron transport between PSII and PSI (Rascher et al., 2010). Taken together, plants produce smaller and thinner leaves under low light conditions, thereby resulting in lower transportation of nutrients, water, and photosynthetic assimilation substrates and ultimately leading to huge losses in the agriculture production (Wang et al., 2020).

To perceive changes in the light environment, plants need to rely on their light signal receptors, such as phytochromes (PHY), cryptochromes (CRY), phototropins (PHOT), and UV Resistance Locus 8 (Voityuk et al., 2014). Phytochrome interacting factors (*PIFs*) are a member of the basic helix-loop-helix (bHLH) transcription factor family, which regulate the transduction of the phytochrome signal pathway (Duek and Fankhauser, 2005). In *Arabidopsis*, the expression levels of *PIF4* and *PIF5* are increased under shading stress (Lorrain et al., 2008). Similar results were found in soybean: The expression level of *GmPIF3a* was significantly upregulated under shading stress (Horvath et al., 2015). Accordingly, plants can sense the changes of external light signals through different photoreceptors and then judge the plant density and the degree of shade stress around themselves. In addition, many phytohormones participate in shade avoidance responses. Auxin (IAA), gibberellins (GAs), brassinolide (BR), and jasmonic acid (JA) are crucial for regulating the shading-induced leaf senescence (Nozue et al., 2015; Jiang et al., 2020). Zhang et al. (2011) showed that under shading stress, abscisic acid (ABA) and zeatin (ZT) concentrations in soybean seedlings decreased compared with normal light treatment, while the concentrations of IAA and GA increased; such a result might be beneficial to reduce the pod abortion. Alterations of light signals under shading stress affect the concentration, transport, and sensitivity of IAA. IAA is the most important plant hormone regulating shade avoidance syndromes (Procko et al., 2014). GAs frequently synergistically work with IAA to promote organ elongation, such as stem and leaf area in shade environment (Alabadí et al., 2004). GA biosynthesis and signaling pathways, such as major GA biosynthetic genes and DELLA proteins, are involved in shading avoidance. Shade-induced stem elongation requires BR as BR biosynthesis mutants were unable to elongate in the shade (Keuskamp et al., 2011). However, the mechanism in which BR regulates the leaf photosynthetic adaptive mechanism is complex, mainly through the interaction with various signal pathways, especially IAA signal pathways (Oh et al., 2014).

Mung bean (*Vigna radiata* L.) is a drought-tolerant crop with a short growth period; it has high protein, medium starch, and low-fat concentrations and is used as food, feed, and medicine (Ganesan and Xu, 2018). Recently, with the adjustment of China’s agricultural structure, mung bean intercropping with other crops, such as proso millet (Gong et al., 2020), cotton (Liang et al., 2020), and oat (Qian et al., 2018), has received increasing attention. However, under an intercropping system, the growth of mung bean plants is obviously affected by shading from high plants in a symbiotic period, leading to weak growth performance and decreased productivity. Therefore, the physiological and ecological response mechanism of mung bean under low light should be explored, and shade-tolerant cultivars should be bred to improve intercropping. To date, many studies about the comprehensive characteristics of soybean (Hussain et al., 2021) and peanut (Wang et al., 2020) under shading stress have been reported. However, few have investigated the responses of mung bean plants under different low-light environments, especially endogenous hormones and gene expression associated with the light intensity. We hypothesized that shading stress would weaken the leaf physiological metabolism by affecting the cell structure and key gene expression, which was ultimately reflected in mung bean plant morphology. The objective of the present study was to investigate the agronomic traits and photosynthetic and Chl fluorescence parameters of mung bean grown under shade environment and further explore the influence mechanism from the internal structure of leaves, endogenous hormone concentration, and key gene expression. Our results suggest that the biosynthesis of endogenous plant hormones play important roles in the fitness and adaption of mung bean plants in response to light intensity-dependent challenges during the cultivation. This study provides insights into the morphological, physiological, and molecular flexibility of mung bean in adapting to the light fluctuation in intercropping or other weak light environments.

## Materials and Methods

### Plant Material and Treatment

The experiment was carried out at the Modern Agricultural Science and Technology Demonstration Park (37°56′26″N, 109°21′46″E), Yulin, Shaanxi, China in 2020. The area has a semi-arid continental monsoon climate with an average annual rainfall of 400 mm and temperature of 10°C. The soil in the 0–20 cm layer at the experimental site had a loess-like loam texture and contained 7.34 g kg^–1^ organic matter, 0.46 g kg^–1^ total nitrogen, 0.75 g kg^–1^ total phosphorus, 32.7 mg kg^–1^ available phosphorus, 17.88 g kg^–1^ total potassium, and 72 mg kg^–1^ available potassium with a pH of 8.6.

This study used two mung bean (*V. radiata* L.) cultivars with different growth periods, namely Xilv 1 (loose-type, the growth period is 85–90 days, plant height is 45 cm, pitch number of main stem are 9–10, pods number per plant are 25–35, identified by the National Identification Committee of Minor Grain Crops, provided by Northwest A&F University) and Yulv 1 (erect-type, the growth period is 90–105 days, plant height is 60 cm, pitch number of main stem are 11–14, pods number per plant are 35–45, bred by Hengshan Daming mung bean system, provided by the Yulin Academy of Agricultural Science). The plants were covered with different densities of polyethylene black shade net to establish the non-shading treatment with natural light conditions of 1,500 μmol m^–2^ s^–1^ (S0), moderate-shading treatment with low-light conditions of 750 μmol m^–2^ s^–1^ (S1), and severe-shading treatment with stress light conditions of 375 μmol m^–2^ s^–1^ (S2). A square iron frame with a height of 2.0 m was used. The light intensity on a sunny day was measured with a light meter (AccuPAR LP-80, WA, United States). Experiments were arranged as a randomized block design with three replicates, and mung beans were planted in the north-south row direction. Each plot area was 10 m^2^ (5 m × 2 m), and mung bean seeds were sowed on May 25 with row spacing of 40 cm and plant spacing of 25 cm. When mung beans grew to the branching stage, shading treatments were imposed. Chemical fertilizer inputs (60 kg N ha^–1^ and 75 kg P_2_O_5_ ha^–1^) were conducted in accordance with the traditional methods of local farmers. During the growth period, no additional fertilizers or irrigations were applied. The mung bean was sampled at 30 days for shading treatments to determine the effects of shading condition on experimental parameters.

The top third fully expanded trifoliate leaves of mung bean were taken at 9:00–11:00 for the determination of physiological and molecular parameters. Leaf samples were immediately homogenized in liquid nitrogen and transported from the field to the laboratory. Then, they were stored at −80°C until use for the evaluation of Chl concentration, enzyme activities, metabolite concentration, plant hormone, and key gene expression. Moreover, the aboveground parts were cut and oven-dried at 75°C for 48 h to measure the biomass. All samples were mixed with three mung beans from the plot, and the measurement was conducted three times.

### Measurement of Microclimate

Illuminance (lux) was measured with a ZDS-10 illuminometer (Shanghai, China), and air temperature and relative humidity were determined using a Hygro-Thermometer Psychrometer (DHM2, Tianjin, China). All measurements were taken from 11:00 to 13:00 on cloudless days.

### Morphological Characteristics

Three mung bean plants from each plot (total 9 plants) were selected randomly to measure agronomic traits. The plant height was measured with a ruler. The stem diameter and first internode length were determined using a vernier caliper. The leaf area was measured with a YMJ-C leaf area meter (Zhejiang, China).^1^ By using software analysis (Intelligent leaf area measurement system, Zhejiang Top Cloud Agricultural Science Co., LTD, Hangzhou, China), the leaf area was obtained by taking pictures of the fresh leaves on a whiteboard.

### Photosynthetic Measurements

The top third fully expanded leaf from each treatment was used to analyze various photosynthetic characteristics with a CIRAS-3 photosynthesis system (PP Systems, Amesbury, MA, United States) at 9:00–11:00. Gas exchange parameters, such as net photosynthetic rate (Pn), transpiration rate (E), stomatal conductance (g*_*s*_*), and intercellular CO_2_ concentration (Ci), were determined under 1,000 μmol m^–2^ s^–1^ light intensity, 400 μmol mol^–1^ atmospheric CO_2_ concentration, and 30°C leaf temperature. Three mung bean plants were used for each plot.

The Chl fluorescence parameters were measured with the MINI-PAM-II fluorometer (Imaging PAM, Walz, Germany). Before measurement, all plants were adapted for 30 min in a dark chamber. The maximal fluorescence of the light-adapted state (Fm) and the maximal PSII quantum yield (Fv/Fm) were determined by a 3,000 ms saturated light pulse. Leaves were illuminated with an actinic light (1,800 μmol (photon) m^–2^ s^–1^). Fv/Fm, photochemical quenching (qP), and non-photochemical quenching (NPQ) were calculated based on the dark- and light-adapted fluorescence measurements in accordance with the method of Gong et al. (2019).

### Chlorophyll Concentration

The total Chl concentration was extracted from frozen samples with 10 ml of 80% acetone in the dark for 24 h. Samples were cut from the middle part with a puncher (1.2 diameter), and the supernatant was measured by a spectrophotometer (UV-2550, Shimadzu, Japan) at wavelengths of 663 and 645 nm to evaluate the Chl *a*, *b*, and total Chl concentration (mg/L). Three plant samples were analyzed in each treatment.

### Measurement of Rubisco and Phosphoenolpyruvate Carboxylase Activities

Fresh samples (0.2 g) were extracted in accordance with the square method of Berveiller and Damesin (2008). The buffer solution was 0.1 mol L^–1^ Tris-HCl (pH 7.4), containing 1.0 mmol⋅L^–1^ ethylenediaminetetraacetic acid (EDTA), 7 mmol L^–1^ mercaptoethyl alcohol, 10% glycerol, and 1% polyvinyl pyrrolidone (PVP). The supernatant was centrifuged at 15,000 × g for 15 min at 4°C to obtain the enzyme extract. The activity of phosphoenolpyruvate carboxylase (PEP Case) (EC 4.1.3) and Rubisco (EC 4.1.1.39) was measured as described by Bi et al. (2015). The absorbance at 340 nm wavelength was traced by using an ultraviolet spectrophotometer, and the enzyme activity was calculated. The concentrations of ATP, starch, sucrose, and soluble sugar in mung bean leaf were determined and calculated in accordance with the method of Li (2000). Each measurement was repeated three times.

### Measurement of Leaf Anatomical Features

Three middle parts of leaves for each treatment without midribs were taken and fixed in formalin-acetic acid-alcohol solution (ethanol:formaldehyde:glacial acetic acid, 90:5:5). The leaf samples were dehydrated in ethanol solutions and then embedded in paraffin. The tissue sections were co-stained by Safranine and Fast Green and observed with a light microscope (ECLIPSE Ts2, Nikon Instruments Inc., Japan).

### Endogenous Hormone Concentration

Leaf tissue (0.1 g fresh mass) was sampled and added to 1 ml extract (acetonitrile:water, 1:1). The supernatants were extracted on ice for 4 h and centrifuged at 4°C for 12,000 × g for 10 min. An aliquot (800 μl) of the supernatant was purified by solid-phase extraction. The solid-phase extraction cartridges were washed using 1 ml of methanol and equilibrated with 1 ml 50% ACN/H_2_O (v/v). The samples were loaded, and then the flow-through fraction was discarded. The cartridge was then rinsed using 1 ml of 60% ACN/H_2_O (v/v). Then, the samples were evaporated to dryness under a gentle stream of nitrogen and reconstituted in 100 μl of 10% ACN/H_2_O (v/v). All the samples were vortexed for 30 s, sonicated in an ice-water bath for 5 min, and then, centrifuged at 4°C for 15 min at 12,000 × g. The ZORBAX Eclipse XDB C18 column (4.6 mm × 280 mm; 5.0 μm) was used to analyze samples. After the crude extract was purified by reverse-phase solid-phase extraction, ether extraction, and derivatization, the endogenous hormone concentration of mung bean leaf was measured by ultra-high-performance liquid chromatography-tandem mass spectrometry (UHPLC-MS/MS, Agilent Technologies, Ltd., Waldbronn, Germany) with Chromosep C18 column (C18 Sep-Pak Cartridge, Waters Corp., Milford, MA, United States) (Ma et al., 2008; Teng et al., 2010).

### Gene Expression

The relative expression of *VrCRY1*, *VrCRY2*, *VrPHYB*, *VrPIF4*, *VrEIN3*, *VrGA2ox*, *VrGA3ox1*, *VrGA20ox1*, *VrROT3*, and *VrBZR1* was assayed with QuantStudio 6 Flex Real-Time PCR System (Thermo Fisher Scientific, MA, United States). The primers for quantitative PCR (qPCR) of genes are shown in Supplementary Table 1. RNA was extracted using the TRIzol™ Plus RNA Purification Kit (Invitrogen, MA, United States). Reverse transcription and amplification of cDNA were performed using SuperScript III First-Strand Synthesis SuperMix for quantitative real-time PCR (qRT-PCR) (Invitrogen).

### Statistical Analysis

ANOVA was conducted to analyze data using SPSS (version 19.0 Chicago, IL, United States). Comparisons among different treatments were based on Duncan’s test at 5% probability level. The significance of treatment effects, varieties, and their interactions were assessed using ANOVA with the standard split-plot design method. All graphs were plotted using Origin 2018.

## Results

### Microclimate in the Field

**FIGURE 1:**
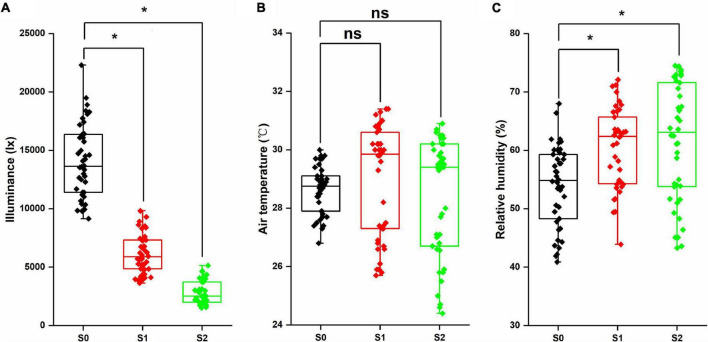
Illuminance **(A)**, air temperature **(B)**, and relative humidity **(C)** in the mung bean field under shading stress. *Significant at 0.05; ns, no significant difference. S0, no shading; S1, moderate shading; and S2, severe shading.

As shown in Figure 1, shading stress significantly decreased the illuminance (*p* < 0.05). Compared with S0, S1, and S2 were reduced by 56.0 and 80.2%, respectively. However, no significant differences were observed in the air temperature between the control and shading stress condition, and the average values of S0, S1, and S2 treatments were 28.5, 28.9, and 28.4°C, respectively. For relative humidity, the shade condition increased by 11.7% in S1 and 13.8% in S2, and the differences were significant between the control and shading stress (*p* < 0.05). Through analyzing microclimate factors, we found that the illuminance and relative humidity were greatly affected by shading stress in the field condition.

### Morphological Characteristics

**FIGURE 2:**
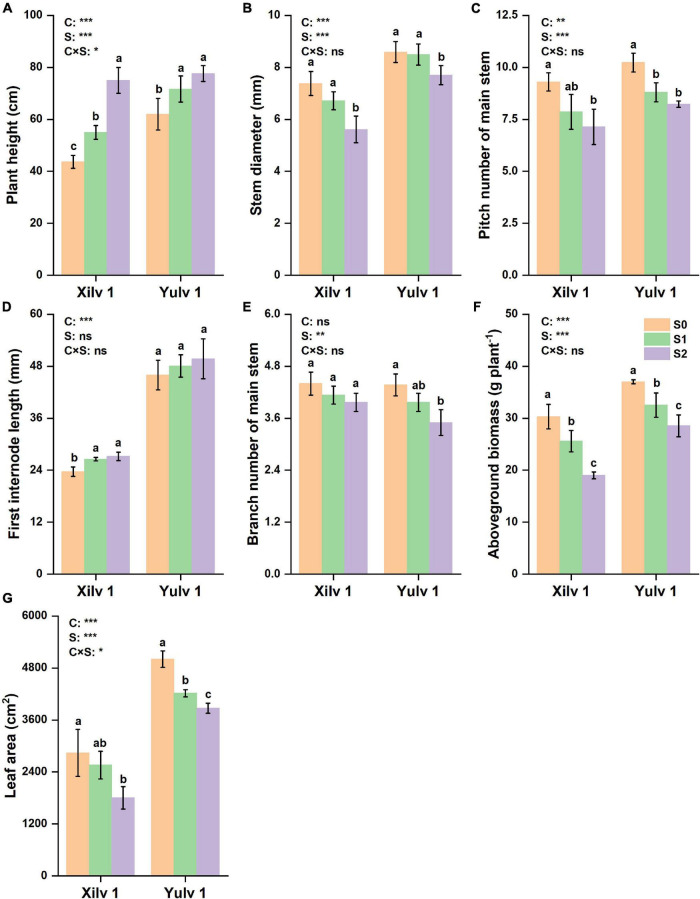
Effect of shading stress on the agronomic traits in mung bean. **(A)** Plant height; **(B)** stem diameter; **(C)** pitch number of main stem; **(D)** first internode length; **(E)** branch number of main stem; **(F)** aboveground biomass; and **(G)** leaf area. Values followed by a different letter are significantly different at *p* < 0.05. S0, no shading; S1, moderate shading; and S2, severe shading. *, **, and *** significant at the 0.05, 0.01, and 0.001 probability levels, respectively. ns, no significant difference.

**FIGURE 3:**
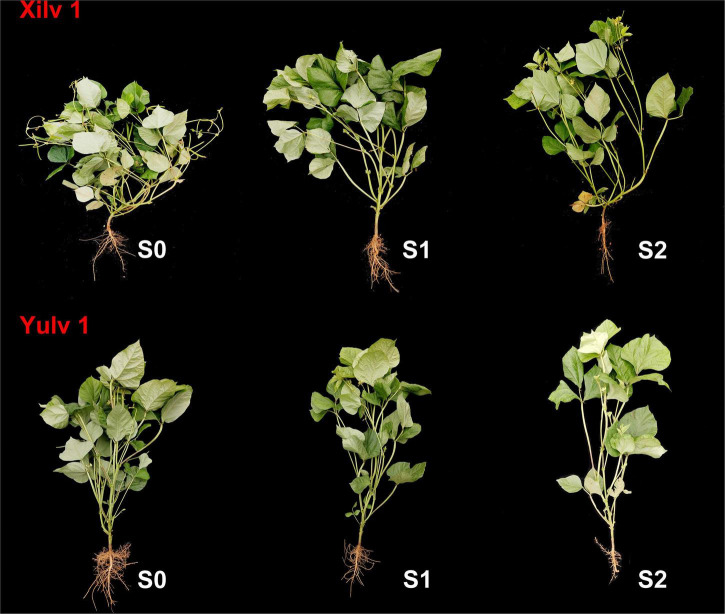
Morphological characteristics of mung bean under shading stress. S0, no shading; S1, moderate shading; and S2, severe shading.

Shading stress had significant effects on the plant morphological characteristics (Figures 2, 3). Plant height and the first internode length were increased, and the stem diameter, pitch number of main stem, branch number of main stem, aboveground biomass, and leaf area were decreased by shading stress, especially in the S2 treatment. For average two shading treatments (S1 and S2), compared with S0, the plant height, stem diameter, pitch number of main stem, first internode length, branch number of main stem, and aboveground biomass of Xilv 1 under low-light condition were influenced by 48.9, 16.5, 19.4, 13.6, 8.0, 26.4, and 23.3%, and those of Yulv 1 were influenced by 20.4, 5.8, 16.8, 6.3, 14.6, 17.5, and 19.2%, respectively. However, the effect of cultivars × shading treatment interaction on the morphological characteristics was not significant (*p* < 0.05) (except for the plant height and leaf area). The results showed that greater morphological plasticity represent greater adaptability to shade.

### Photosynthetic Parameters

**FIGURE 4:**
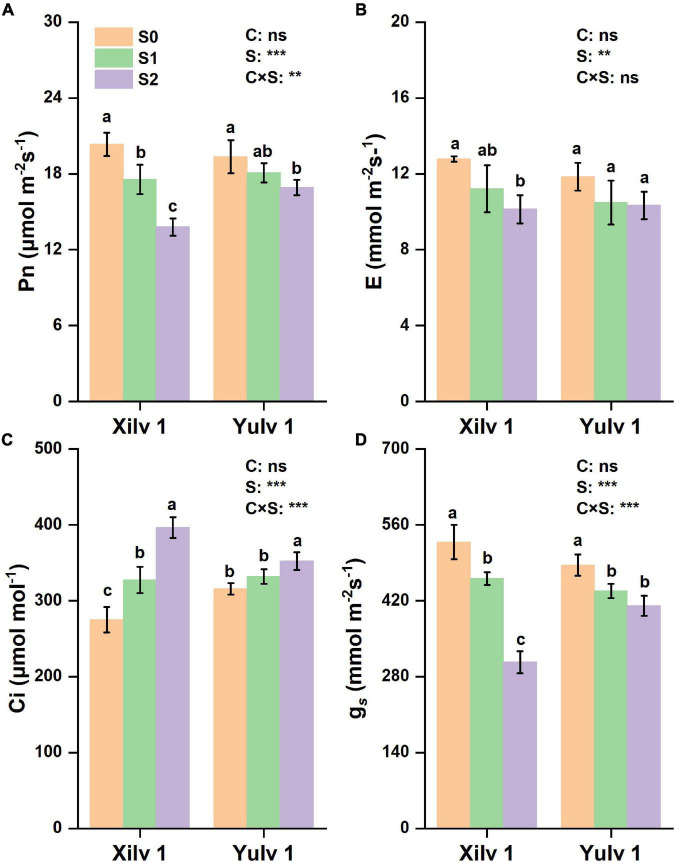
Effect of shading stress on the leaf gas exchange parameters in mung bean. **(A)** Net photosynthetic rate; **(B)** transpiration rate; **(C)** intercellular CO_2_ concentration; and **(D)** stomatal conductance. Values followed by a different letter are significantly different at *p* < 0.05. S0, no shading; S1, moderate shading; S2, severe shading. ** and *** significant at the 0.01 and 0.001 probability levels, respectively. ns, no significant difference.

The gas exchange parameters of the two mung bean cultivars are shown in Figure 4. The net photosynthetic rate (Pn), transpiration rate (E), and stomatal conductance (g*_*s*_*) were significantly decreased by 22.9, 16.5, and 27.4% in Xilv 1 and 9.6, 12.1, and 12.6% (*p* < 0.05) in Yulv 1 under shading stress (averaged S1 and S2) compared with the control. Greater reduction of photosynthetic parameters was observed in S2 treatment than in S1 treatment. By contrast, the shading stress significantly increased intercellular CO_2_ concentration (Ci) of leaves (31.4 and 8.4% in Xilv 1 and Yulv 1, respectively; *p* < 0.05). Yulv 1 had higher photosynthetic capacity, indicating that this cultivar has strong shade tolerance.

**FIGURE 5:**
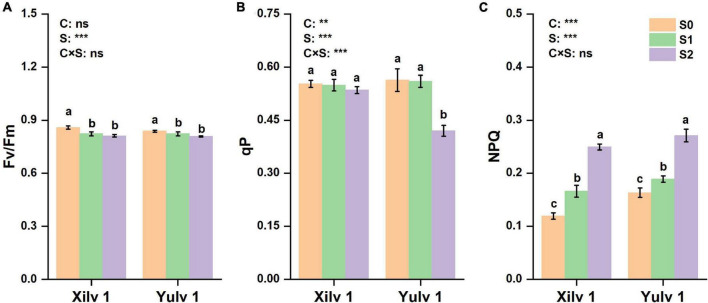
Effect of shading stress on the leaf chlorophyll (Chl) fluorescence parameters in mung bean. **(A)** The maximal photosystem II (PSII) quantum yield; **(B)** photochemical quenching; and **(C)** non-photochemical quenching (NPQ). Values followed by a different letter are significantly different at *p* < 0.05. S0, no shading; S1, moderate shading; and S2, severe shading. ** and *** significant at the 0.01 and 0.001 probability levels, respectively. ns, no significant difference.

Similarly, the Chl fluorescence parameters, such as maximal PSII quantum yield (Fv/Fm), photochemical quenching (qP), and non-photochemical quenching (NPQ) were determined (Figure 5). Fv/Fm was significantly decreased by 4.9% in Xilv 1 and 2.7% (*p* < 0.05) in Yulv 1 under shading stress compared with control. No significant differences were observed between S1 and S2 treatment. Furthermore, only S2 treatment significantly decreased qP of Yulv 1 (25.4% lower over normal light control) (*p* < 0.05). Simultaneously, S1 and S2 treatments induced the increase of NPQ in Xilv 1 and Yulv 1 in the field condition.

### Enzymatic Activity and Assimilation Substances

**FIGURE 6:**
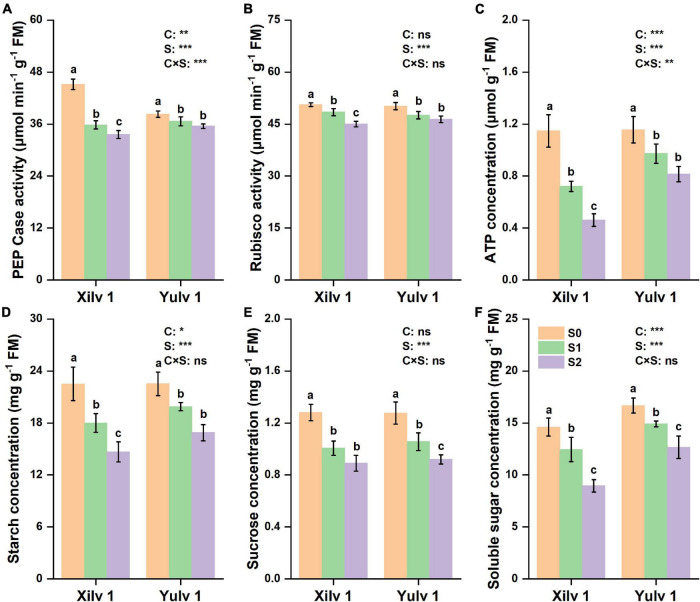
Effect of shading stress on the leaf physiological parameters in mung bean. **(A)** Phosphoenolpyruvate carboxylase (PEP Case); **(B)** Rubisco; **(C)** ATP; **(D)** starch; **(E)** sucrose; and **(F)** soluble sugar. Values followed by a different letter are significantly different at *p* < 0.05. S0, no shading; S1, moderate shading; and S2, severe shading. *, **, and *** significant at the 0.05, 0.01, and 0.001 probability levels, respectively. ns, no significant difference.

In the present study, significant differences were observed in PEP Case, Rubisco, ATP, starch, sucrose, and soluble sugar concentrations at different light intensity treatments (Figure 6). Shading stress significantly decreased the activity of PEP Case and Rubisco and concentrations of ATP, starch, sucrose, and soluble sugar. On average, the reduction under weak light stress was 14.5, 7.0, 20.5, 22.9, 24.2, and 22.0% (*p* < 0.05), respectively, compared with normal light control (S0). Yulv 1 had strong shade tolerance than Xilv 1, as the decrease of PEP Case, Rubisco, ATP, starch, sucrose, and soluble sugar of Yulv 1 (5.9, 6.4, 22.7, 18.4, 22.6, and 17.3%) under low-light and shading stress was less than that of Xilv 1 (23.2, 7.7, 48.5, 27.4, 25.9, and 26.7%). The effect of cultivars on the enzymatic activity and assimilation substances was significant (*p* < 0.05) (except for Rubisco activity and sucrose concentration).

### Chlorophyll Concentration and Leaf Anatomical Structure

**FIGURE 7:**
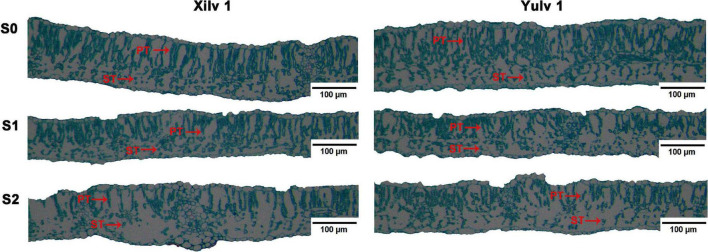
Effect of shading stress on the leaf anatomical features in mung bean. ST, spongy tissues; PT, palisade tissues. S0, no shading; S1, moderate shading; and S2, severe shading.

Different light conditions had a significant effect on the Chl concentration of mung bean plants (Table 1). The concentrations of Chl *a*, Chl *b*, and Chl (*a* + *b*) of both cultivars under shading stress increased remarkably compared with those in control, while decreased Chl *a/b* was found (*p* < 0.05). Light quantity strongly affects the leaf anatomy (Figure 7). We found that in S0 treatment, the mung bean leaf showed clear and compact tissue structure. With decreasing light intensity from S1 to S2, the palisade tissues and spongy tissues were seriously thinner. In Xilv 1, the leaf tissue arrangement was loose and scattered, the shape was irregular, and the gap was large. These findings indicated that shading stress negatively influenced the mung bean leaf tissue size.

**TABLE 1:** Effect of shading stress on the leaf chlorophyll concentration in mung bean.

Cultivar	Treatment	Chl *a* (mg g^–1^)	Chl *b* (mg g^–1^)	Chl *a* + *b* (mg g^–1^)	Chl *a/b*
Xilv 1	S0	0.23 ± 0.02b	0.12 ± 0.01c	0.35 ± 0.03b	1.99 ± 0.12a
	S1	0.24 ± 0.01b	0.14 ± 0.01b	0.39 ± 0.01b	1.73 ± 0.07b
	S2	0.30 ± 0.01a	0.18 ± 0.01a	0.48 ± 0.01a	1.63 ± 0.05b
Yulv 1	S0	0.37 ± 0.01b	0.13 ± 0.01	0.50 ± 0.02c	2.95 ± 0.11a
	S1	0.39 ± 0.01a	0.16 ± 0.01b	0.55 ± 0.01b	2.52 ± 0.06b
	S2	0.41 ± 0.01a	0.18 ± 0.01a	0.59 ± 0.01a	2.27 ± 0.10c
**Variation source**					
Cultivar (C)		***	*	***	***
Treatment (T)		***	***	***	ns
C × T		ns	ns	*	ns

Values followed by a different letter within the same column are significantly different at p < 0.05. * and *** significant at the 0.05 and 0.001 probability levels, respectively. ns, no significant difference.

### Endogenous Hormone Concentration and Key Gene Expression

As shown in Table 2, the endogenous hormone concentration of mung bean leaf was strongly affected by shading stress. Specifically, the concentrations of auxin (IAA, indole-3-acetic acid), ABA, GAs (such as, GA1, GA3, and GA7), and BR in both cultivars significantly increased by 36.5, 79.6, 55.1, and 31.1% under shading conditions (*p* < 0.05), while the concentrations of salicylic acid (SA) and ZT significantly decreased by 62.1 and 24.6% (*p* < 0.05). Interestingly, the opposite trend was observed in the JA concentration of both cultivars under shading conditions: the JA concentration in Xilv 1 was improved, whereas that in Yulv 1 was reduced. In addition, compared with S0, the variation range of IAA, ABA, GA, BR, SA, Zt, and JA concentrations in Xilv 1 under shading stress was 36.6, 131.9, 53.7, 43.2, 62.6, 17.8, and 106.8% (*p* < 0.05); by contrast, that in Yulv 1 was 35.9, 19.8, 57.1, 13.1, 61.5, 35.8, and 31.3% (*p* < 0.05), respectively. The effect of cultivars on the endogenous hormone concentration was significant (*p* < 0.05) (except for GA3 and JA). Through analyzing the variation of endogenous hormone concentration, Xilv 1 was more sensitive and changed more than Yulv 1 under shading stress.

**TABLE 2:** Effect of shading stress on the leaf hormone concentrations (ng g^–1^, FM) in mung bean.

Cultivar	Treatment	IAA	ABA	GA1	GA3	GA7	SA	JA	BR	ZT
Xilv 1	S0	3.88 ± 0.13c	2.71 ± 0.12c	0.34 ± 0.03c	0.35 ± 0.04b	0.11 ± 0.01b	154.60 ± 5.48a	7.40 ± 0.35c	0.13 ± 0.01c	0.30 ± 0.01a
	S1	6.02 ± 0.16a	5.81 ± 0.34b	0.39 ± 0.02b	0.66 ± 0.05a	0.26 ± 0.03a	64.94 ± 5.05b	13.53 ± 1.02b	0.17 ± 0.01b	0.27 ± 0.01b
	S2	4.58 ± 0.29b	6.75 ± 0.16a	0.44 ± 0.02a	0.42 ± 0.03b	0.28 ± 0.01a	50.51 ± 3.69c	17.09 ± 0.82a	0.21 ± 0.01a	0.23 ± 0.01c
Yulv 1	S0	1.21 ± 0.13b	2.37 ± 0.16b	0.04 ± 0.00b	0.36 ± 0.03c	0.13 ± 0.00b	135.38 ± 4.97a	16.27 ± 1.39a	0.09 ± 0.00b	0.18 ± 0.02a
	S1	1.72 ± 0.16a	2.78 ± 0.17a	0.12 ± 0.01a	0.50 ± 0.03b	0.15 ± 0.01b	54.41 ± 4.28b	14.96 ± 0.99a	0.10 ± 0.01ab	0.13 ± 0.01b
	S2	1.57 ± 0.09a	2.89 ± 0.18a	0.11 ± 0.01a	0.59 ± 0.04a	0.21 ± 0.02a	49.80 ± 2.59b	7.38 ± 0.51b	0.11 ± 0.01a	0.10 ± 0.01b
**Variation source**										
Cultivar (C)		***	***	***	ns	***	***	ns	***	***
Treatment (T)		***	***	***	***	***	***	***	***	***
C × T		***	***	ns	***	***	*	***	***	ns

IAA, indole-3-acetic acid; ABA, abscisic acid; GA1, gibberellin A1; GA3, gibberellin A3; GA7, gibberellin A7; SA, salicylic acid; JA, jasmonic acid; BR, brassinolide; ZT, zeatin. Values followed by a different letter within the same column are significantly different at p < 0.05. * and *** significant at the 0.05 and 0.001 probability levels, respectively. ns, no significant difference.

**FIGURE 8:**
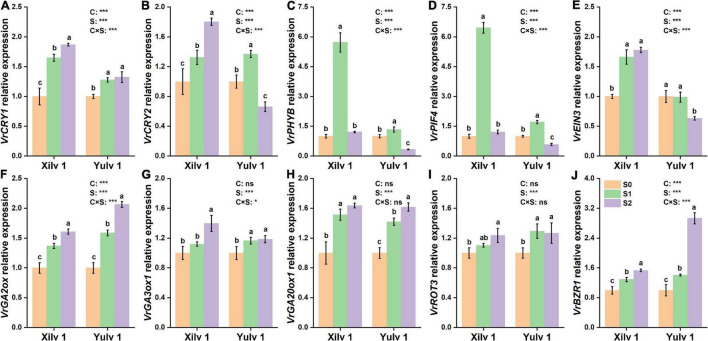
Effect of shading stress on the relative expressions of key leaf genes in mung bean. **(A)** *VrCRY1*; **(B)** *VrCRY2*; **(C)** *VrPHYB*; **(D)** *VrPIF4*; **(E)** *VrEIN3*; **(F)** *VrGA2ox*; **(G)**, *VrGA3ox1*; **(H)** *VrGA20ox1*; **(I)** *VrROT3*; and **(J)** *VrBZR1*. Values followed by a different letter are significantly different at *p* < 0.05. S0, no shading; S1, moderate shading; and S2, severe shading. * and *** significant at the 0.05 and 0.001 probability levels, respectively. ns, no significant difference.

Furthermore, the expressions of two genes involved in cryptochromes (*VrCRY1* and *VrCRY2*), two genes involved in phytochromes (*VrPHYB* and *VrPIF4*), one gene involved in ethylene biosynthesis (*VrEIN3*), three genes involved in GA biosynthesis (*VrGA2ox*, *VrGA3ox1*, and *VrGA20ox1*), and two genes involved in BR biosynthesis (*VrROT3* and *VrBZR1*) were quantitatively analyzed (Figure 8). The relative expressions of *VrCRY1*, *VrCRY2*, *VrPHYB*, *VrPIF4*, and *VrEIN3* in Xilv 1 were remarkably upregulated by shading stress compared with those in control (*p* < 0.05). However, for Yulv 1, the gene expression levels under two shading stresses showed different trends. In addition, in endogenous hormone biosynthesis, the relative expression levels of all five genes for GA (3) and BR (2) were upregulated under low-light condition (*p* < 0.05). The expression levels of *VrGA2ox*, *VrGA3ox1*, *VrGA20ox1*, *VrROT3*, and *VrBZR1* in Xilv 1 and Yulv 1 were increased by 1. 49-, 1. 26-, 1. 58-, 1. 17-, and 1.41-fold and 1.83, 1.18, 1.52, 1.28, and 2.17-fold, respectively, in shading stress compared with the S0 treatment. Furthermore, the effect of cultivars × shading treatment interaction on the gene expression was significant (*p* < 0.05) (except for *VrGA20ox1* and *VrROT3*).

## Discussion

### Responses of Agronomic Traits, Photosynthetic Characteristics, and Chlorophyll Fluorescence Parameters to Shading Stress: From Morphology to Physiology

Crop morphology has a certain degree of plasticity, and there is a corresponding adaptation mechanism to different ecological environments (Gong et al., 2015). Many studies have shown that shading promotes the upward growth of stems and petioles but reduces the leaf area of plants (Liu et al., 2017; Jiang et al., 2020). Similar results were observed in our experiment. The plant height and first internode length of mung bean significantly increased under shading conditions, while the stem diameter, pitch number of main stem, and branch number of main stem significantly decreased, resulting in a reduction in aboveground biomass (Figure 2). The lower the light intensity, the greater the influence of the value. These features indicated that the changes in light environment would affect the morphological parameters of mung bean, and in turn, these morphological changes could induce plants to absorb more light energy and reduce the shading stress. Meanwhile, shading not only reduced the light intensity of the environment, but also increased the relative humidity, as shown in Figure 1. This phenomenon may lead to an increase in the occurrence of diseases and insect pests and further weaken the physiological metabolic ability. Crop leaves are sensitive to the light environment, and the photosynthetic capacity is affected by shading stress to a certain extent (Iram et al., 2021). The net photosynthetic rate, transpiration rate, and stomatal conductance were significantly decreased by 16.3, 14.3, and 20.0%, respectively, under shading stress compared with control (Figure 4). By contrast, shading stress significantly increased the intercellular CO_2_ concentration of leaves by 19.9% (*p* < 0.05), indicating that shading stress weakened the net photosynthetic rate of mung bean leaves by non-stomatal limitation. This effect may be associated with the leaf structure changes under shading stress. Lower light environment induced large cell gap, loose cell arrangement, and decreased palisade and spongy tissue thickness in leaves (Figure 7), resulting in decreased chloroplast channel area through which carbon dioxide enters. Consequently, the thickness of leaves and photosynthetic capacity of mung bean leaves are significantly weakened (Feng et al., 2019).

**FIGURE 9:**
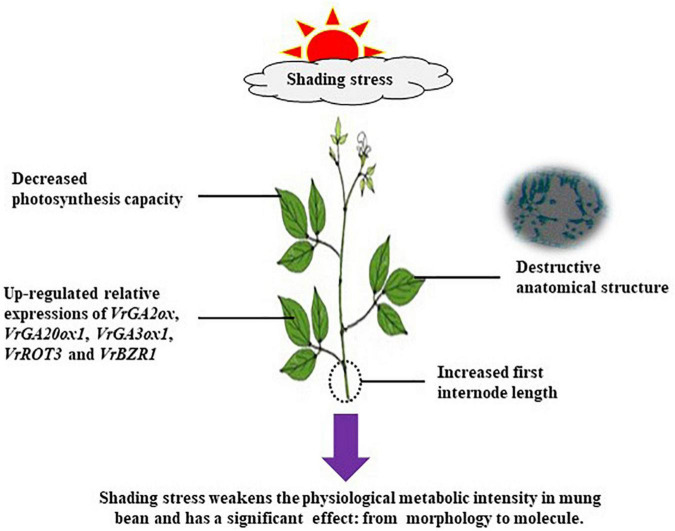
Schematic representation of changes in morphological characteristics, leaf physiology, and photosynthetic characteristics of mung bean plants as affected by shading stress.

As an internal probe, Chl fluorescence is widely applied in crop science to analyze the relationship between abiotic stress and photosynthetic capacity (Dai et al., 2009). In the current study, the photosynthesis of mung bean was severely damaged by shading stress as shown by the Chl fluorescence parameters (Figure 5). Increased NPQ indicated that the absorbed energy of PSII flux to photochemical processes was reduced under shading stress, and this part of energy was converted into non-photochemical energy loss as heat (Li et al., 2021). However, Fv/Fm was not functionally impaired, even with a significant decrease. The possible reason was that the higher PSII/PSI ratio contributed to compensate for the reduction in the amount of red light, which is required to excite PSII (Iram et al., 2021). Such results may suggest that vulnerability to a lower photosynthetic rate might be linked with changes in multiprotein complexes (PSI and PSII) (Muneer et al., 2014). Moreover, these changes in Chl fluorescence parameters were strongly related to the decreased photosynthetic rate as suggested earlier. Chl can absorb, transmit, and convert light energy and is an important part of the plant photosynthetic system (Gill and Tuteja, 2010). Shading stress increased the Chl concentration in mung bean leaf, such as Chl *a*, Chl *b*, and total Chl *a* + *b* (Table 1). This phenomenon was beneficial to absorb and capture more light energy from the low-light environment and improve the efficiency of light energy utilization (Wang et al., 2020). However, decreased Chl *a/b* was mainly observed because shading stress induced the increase in Chl *b* concentration than Chl *a* concentration. Studies have demonstrated that Chl *a* is directly associated with the leaf photosynthesis capacity, while Chl *b* is located in the photochromatin complex of PSII for trapping diffuse light (Field et al., 2013; Yao et al., 2017b). Increased Chl *b* concentration can play an important role in enhancing the survival ability of mung bean under shading environment. Furthermore, we investigated the activities of related photosynthetic enzymes and assimilate metabolism. Our results showed that shading stress significantly decreased the concentrations of starch, sucrose, and soluble sugar in the leaves of mung bean compared with control, ultimately leading to the decline in ATP concentration (Figure 6). These results are consistent with the findings of Hussain et al. (2019), suggesting that anatomical and biochemical changes in shaded plants represent ways to cope with low light availability and energy cost of re-fixing CO_2_ induced by leakiness under shading (Amiard et al., 2005; Feng et al., 2019). Although the activities of PEP Case and Rubisco were significantly reduced, the diminutive decline may be negligible to a certain extent. Hence, the performances in photosynthetic enzyme activities in shaded plants were potential physiological strategies for decreasing the energy cost under low light availability and keeping the equilibrium of metabolites and energy fluxes between mesophyll and bundle sheath cells (Sales et al., 2018). Taken together, shading remarkably reduced the net photosynthetic rate of mung bean leaves. On the one hand, this phenomenon was attributed to the destruction of the internal structure, such as palisade and spongy tissues; on the other hand, it was significantly related to the Chl and light energy utilization, thereby resulting in the decrease of photosynthetic capacity and changes in morphological parameters of mung bean (Figure 9).

### Responses of Endogenous Hormone Concentration and Key Gene Expression to Shading Stress: From Physiology to Molecule

As information transmitters, plant hormones play an important role in regulating the adaptive response of plants. In *Arabidopsis* seedlings, IAA, GAs, and BR regulate hypocotyl and petiole elongation (Yang and Li, 2017). In the present study, the increments in the concentrations of IAA, ABA, GAs, and BR were observed under shading stress (Table 2). Due to weak light, a large amount of IAA was synthesized in the leaves of mung bean, which was transported to the stem and acted on the epidermal cells, thus promoting the over-elongation of plant height (Procko et al., 2014). The changes of optical signal can directly affect the concentration of IAA through *PIFs*. *PIF4* is one of the most important factors that regulates IAA biosynthesis by binding the promoter of key IAA biosynthesis enzymes *YUCs* and triggers shade avoidance (Keuskamp et al., 2010; Hornitschek et al., 2012). For the transport of IAA, excessive IAA produced by shading stress upregulated the relative expression of *VrPIF4* (Figure 8), so that IAA was transported to the growing center more quickly.

Gibberellins are a kind of hormones that regulate the plant growth and development. Among the physiologically active GAs, GA1, and GA4 are involved in plant photomorphogenesis and closely related to light signals (Alabadí et al., 2004). The bioactive GA in plants is mainly regulated by the syngenetic genes *GA20ox* and *GA3ox* and decomposing gene *GA2ox* (Reinecke et al., 2013). GAs promoted cell elongation by changing the rheological properties of the cell wall. The expression of these genes could regulate the concentration of GAs and then affect stem elongation. Under shading stress, the relative expressions of *VrGA2ox*, *VrGA20ox1*, and *VrGA3ox1* related to GA synthesis were increased (Figure 8); the concentrations of GA1, GA3, and GA7 in mung bean were significantly improved (Table 2); and the sensitivity of plants to GAs was obviously enhanced. Genetic evidence showed that GAs stimulated cell elongation by destabilizing the GA signaling repressor DELLA proteins, thus releasing the DNA-recognition domain of the transcription factor (Achard et al., 2007).

Brassinolide is a known steroidal plant hormone, which is important for hypocotyl elongation. The relative expression of BR biosynthesis gene *VrROT3* was increased under shade conditions, further enhancing BR biosynthesis and promoting *VrBZR1* transcription (Figure 8). *VrBZR1* and *PIFs* are interdependent in the regulation of hypocotyl growth, and the degradation of *VrPIF4* or the reduction of *VrBZR1* activity weakened their functions (Oh et al., 2012). The simultaneous application of IAA and BR had a superimposed effect on promoting axial growth of the lower embryo in hormone interaction (Keuskamp et al., 2011). Brassinosteroid-insensitive 2 in the BR signaling pathway could reduce the DNA binding ability and transcriptional inhibition ability of IAA transcription factor repressor ARF2 through oxidative phosphorylation, thus enabling activated auxin response factors (ARFs) to regulate the expression of downstream genes (Vert et al., 2008). In addition, shading treatment altered the levels of other hormones, for example, increased ABA and decreased SA and ZT concentrations (Table 2). These observations are similar to those of previous studies (Li et al., 2019; Jiang et al., 2021). ABA mainly inhibits the cell division and elongation and affects the growth of plant organs. The increase of ABA concentration in leaves indicated that the senescence process of mung bean was accelerated under shading. ZT is a kind of cytokinin that enlarges cell volume by promoting the cell lateral growth. The changes of ZT concentration under shading were significantly related to the downregulation of ZT synthesis genes or upregulation of ZT degradation genes (Roman et al., 2016). Therefore, these hormonal trends may be a way for leaves to respond to low light stress.

### Yulv 1 Has Stronger Tolerance and Ecological Adaptability Than Xilv 1 to Shading Stress

Light is not only the driving force of plant photosynthesis, but also the signal of plant morphological and physiological adaptation to environmental changes (Jiang et al., 2021). In our study, although Xilv 1 showed a higher increase rate in the plant height and first internode length than Yulv 1 under shading stress, Xilv 1 showed lower aboveground biomass than Yulv 1 (Figure 2). This result could be associated with the management of photosynthate allocation under shading stress. Xilv 1 allocated more photosynthetic products to the elongation of the main stem to obtain more light. Meanwhile, the larger leaf area of Yulv 1 is a potential strategy for maintaining photosynthesis (Figure 2). The thylakoid structure and photosynthetic pigment biosynthesis affect the photosynthetic capacity (Yang et al., 2018b). Lower light intensity decreased leaf thickness, palisade tissues thickness, and spongy tissues thickness of leaves. However, compared with Xilv 1, Yulv 1 still maintained a normal cell structure or had less damage to shading stress (Figure 7). This phenomenon might be due to the cell growth and cell layer number in palisade tissues (Kalve et al., 2014). Furthermore, Yulv 1 had higher Chl concentrations (Chl *a*, Chl *b*, and Chl *a* + *b*) than Xilv 1 under shading stress (Table 1), the effect of cultivars on the Chl concentration was significant (*p* < 0.05), indicating that Yulv 1 has stronger tolerance to shading, because increased Chl concentrations under shading conditions, especially the Chl *b*, were beneficial for enhancing the light harvesting in shade-tolerant varieties (Valladares and Niinemets, 2008), as reported by Zhu et al. (2008) that low light-tolerant hybrid rice exhibited a higher content of Chl *b* when exposed to low light. This conclusion was directly supported by the higher photosynthetic assimilation substances and enzyme activities (Figure 6). This strong physiological metabolism strategy may be one of the potential mechanisms explaining why Yulv 1 maintained higher shading resistance levels than Xilv 1.

The variable light environment regulates the expression of related genes to improve the fitness (Jiang et al., 2020). *PIFs* are a kind of transcription factor that directly interacts with the photosensitive chromatin downstream. *PIF4*, *PIF5*, and *PIF7*, the members of the PIF family, are involved in the response to shading stress, and *PIF4*/*PIF5* double mutants and *PIF7* mutants are significantly inhibited in hypocotyl elongation under low-light environments (Leivar and Quail, 2011; Li et al., 2012). Our data showed that shading increased the relative expression of *VrPIF4* in Xilv 1. However, in Yulv 1, moderate shading (S1) treatment significantly upregulated the relative expression of *VrPIF4*, and the opposite trend was observed in severe stress (S2) treatment (Figure 8). Similar results were found in *VrCRY2* and *VrPHYB* under shading condition. As a photosensitive interaction factor, *VrPIF4* can interact not only with photosensitive *VrPHYB*, but also with cryptochrome *VrCRY2*. The changes of these genes indicated that the photoreceptors of Xilv 1 were more responsive to shading than those of Yulv 1, and the downregulated expressions of *VrCRY2*, *VrPHYB*, and *VrPIF4* were beneficial to maintain the plant physiological metabolism of Yulv 1 and ensure plant growth. In addition, the transcription factor *EIN3* in the ethylene signaling pathway has been shown to bind to the promoter of *PIF3*, thereby promoting the expression of *PIF3* and regulating hypocotyl length (Zhong et al., 2012). The upregulated relative expression of *VrEIN3* in mung bean leaves under shade condition led to the accelerated plant senescence, which further supported the conclusion that Yulv 1 has stronger tolerance and ecological adaptability to shading stress than Xilv 1. However, the current results are limited, mainly because it was only a 1-year field experiment. To make the results more convincing, we will conduct multi-year or greenhouse studies. In addition, a follow-up study (i.e., transcriptomics, metabonomics, and proteomics) should be conducted to obtain further insights regarding the mechanisms underlying the tolerance advantages. Meanwhile, an effective potential strategy for enhancing the plant tolerance and mitigating injure from shading stress should be exported in the future sustainable agricultural production.

## Conclusion

Our study found that the mung bean morphological traits and physiological metabolism capacities were changed in response to shading stress. The reduced parameters of plants can be explained by leaf anatomical structure with destroyed palisade and spongy tissues and decreased Chl *a* and *b* concentrations. The upregulated relative expressions of genes induced by reduced weak light intensity are mainly enriched in plant hormone signal transductions. Combined with the physiological and biochemical metabolism, Yulv 1 has stronger tolerance and ecological adaptability to shading stress than Xilv 1. Our results provide insights into the plant mechanisms in response to shading stress, and these parameters could be used to evaluate mung bean cultivars for shading tolerance.

## Data Availability Statement

The original contributions presented in the study are included in the article/Supplementary Material, further inquiries can be directed to the corresponding author/s.

## Author Contributions

XG, CL, and HW performed most of the experiment, analyzed the data, and completed the first draft. XG, BF, and YJ designed the experimental plan and edited the manuscript. CL worked with KD, WD, and HQ to provide suggestions for data analysis and manuscript writing. All authors read and approved the final manuscript.

## Conflict of Interest

The authors declare that the research was conducted in the absence of any commercial or financial relationships that could be construed as a potential conflict of interest.

## Publisher’s Note

All claims expressed in this article are solely those of the authors and do not necessarily represent those of their affiliated organizations, or those of the publisher, the editors and the reviewers. Any product that may be evaluated in this article, or claim that may be made by its manufacturer, is not guaranteed or endorsed by the publisher.

## Footnotes

1
https://www.ybzhan.cn/chanpin/11200164.html
