# Supplementary material for: Mung Bean (Vigna radiata L.) Source Leaf Adaptation to Shading Stress Affects Not Only Photosynthetic Physiology Metabolism but Also Control of Key Gene Expression
Source: Front Plant Sci. 2022 Feb 4;13:753264. doi: 10.3389/fpls.2022.753264 (PMC8854224; doi:10.3389/fpls.2022.753264)
Supplement: Supplementary file 1 [file Table_1.docx]

**TABLE S1** Mung bean primer information.

| **Gene ID** | **Primer sequence** |
| --- | --- |
| *VrCRY1* | 5’−GGGAGGGTTTCTAGGTGGTG−3’ |
|  | 5’−ACGGACGGTAATGCCTTGTG−3’ |
| *VrCRY2* | 5’−CTCTTGCTCACCTGGATCGG−3’ |
|  | 5’−GCACAGAGATGCCTTGTTCC−3’ |
| *VrPHYB* | 5’−CGGATGGGCTCACTCTTCTC−3’ |
|  | 5’−GCGTCACAGGGAGTTCAAGA−3’ |
| *VrPIF4* | 5’−CGTTCCAGAAGAAACCGTGC−3’ |
|  | 5’−AGCCCCCATGCACATTACC−3’ |
| *VrEIN3* | 5’−ACCCAGTCCTTTGTTCCACC−3’ |
|  | 5’−TGTCGAAGGCAGGGATTCTC−3’ |
| *VrGA2ox* | 5’−CTGTCTCGCAGATGGCACTT−3’ |
|  | 5’−CACTCAAAGCTGGTCCTCCAA−3’ |
| *VrGA3ox1* | 5’−CACGGACTCCACCCTTTTGA−3’ |
|  | 5’−CATAGATAAGCAACCGAAAGCCT−3’ |
| *VrGA20ox1* | 5’−AACCTGACCTGACTTTGGGC−3’ |
|  | 5’−ACTGCCCTATGCAAGCAACT−3’ |
| *VrROT3* | 5’−TCCTCCGATCCCCACAACT−3’ |
|  | 5’−CTTCACCAGGACCAACGCTC−3’ |
| *VrBZR1* | 5’−ACATTGCGACAACAACGAGG−3’ |
|  | 5’−TCGGAAATGACGACGAGAGG−3’ |
